# *ZOO*: an automatic data-collection system for high-throughput structure analysis in protein microcrystallography

**DOI:** 10.1107/S2059798318017795

**Published:** 2019-01-28

**Authors:** Kunio Hirata, Keitaro Yamashita, Go Ueno, Yoshiaki Kawano, Kazuya Hasegawa, Takashi Kumasaka, Masaki Yamamoto

**Affiliations:** a RIKEN SPring-8 Center, 1-1-1 Kouto, Sayo, Hyogo 679-5148, Japan; b Japan Science and Technology Agency, Precursory Research for Embryonic Science and Technology (PRESTO), 4-1-8 Honcho, Kawaguchi, Saitama 332-0012, Japan; c Japan Synchrotron Radiation Research Institute, 1-1-1 Kouto, Sayo 679-5198, Hyogo, Japan

**Keywords:** protein microcrystallography, automated data collection, *ZOO*

## Abstract

Owing to the development of brilliant microfocus beamlines, rapid-readout detectors and sample changers, protein microcrystallography is rapidly becoming a popular technique for accessing structural information from complex biological samples. However, the method is time-consuming and labor-intensive and requires technical expertise to obtain high-resolution protein crystal structures. At SPring-8, an automated data-collection system named *ZOO* has been developed. This system enables faster data collection, facilitates advanced data-collection and data-processing techniques, and permits the collection of higher quality data. In this paper, the key features of the functionality put in place on the SPring-8 microbeam beamline BL32XU are described and the major advantages of this system are outlined. The *ZOO* system will be a major driving force in the evolution of the macromolecular crystallography beamlines at SPring-8.

## Abstract 2

An automated data-collection system named *ZOO* has been developed. This system enabled faster data collection, facilitated advanced data-collection and data-processing techniques, and permitted the collection of higher quality data.

## 1. Introduction   

The elucidation of high-resolution structures of biological macromolecules has contributed greatly to our understanding of biological processes at the molecular level. Although macromolecular crystallography is a powerful technique, it is sometimes difficult to crystallize important targets, such as macromolecular complexes and membrane proteins, for basic and applied sciences. Recently, the small and brilliant X-ray beams that are now available at synchrotron facilities have enabled the structural analyses of difficult proteins, even when only poorly diffracting crystals are available (Smith *et al.*, 2012 ▸).

Depending on the available beam and crystal size, there are several experimental schemes that are suitable for individual samples. For crystals grown by the *in meso* method, the multiple small-wedge scheme, which was originally reported for the high-resolution structural analysis of G-protein-coupled receptors (GPCRs), is a useful approach that can be performed at cryogenic temperatures (Cherezov *et al.*, 2007 ▸; Rasmussen *et al.*, 2012 ▸; Rosenbaum *et al.*, 2012 ▸). In this scheme, X-ray-based raster scanning is applied to a cryoloop containing several tens of crystals to specify each crystal position (Cherezov *et al.*, 2009 ▸). Small-wedge data, typically 5–10°, are then collected from each crystal. The process is repeated for a number of sample loops in order to obtain sufficient amounts of data to form a complete data set. The helical data-collection scheme (Flot *et al.*, 2010 ▸) is also a reasonable approach for larger crystals. The scheme enables the distribution of the absorbed dose equally over the entire crystal volume by changing the position of X-ray irradiation during the rotation measurement. *De novo* structural determination of *in meso* 15 µm crystals by combining the helical scheme and microfocused beams has shown tremendous potential (Kumazaki *et al.*, 2014 ▸; Yamamoto *et al.*, 2017 ▸).

Recently, major advancements have been made in serial crystallography using X-ray free-electron lasers (Chapman *et al.*, 2011 ▸; Schlichting, 2015 ▸). For example, various techniques for delivering many thousands of crystals to the X-ray free-electron laser position (Gati *et al.*, 2014 ▸; Coquelle *et al.*, 2015 ▸; Weinert *et al.*, 2017 ▸; Roedig *et al.*, 2017 ▸) and methods for processing large numbers of still diffraction frames (White *et al.*, 2013 ▸, 2016 ▸) have been developed. Consequently, these techniques have inspired the development of serial experiments at synchrotron facilities, including continuous flows of crystals (Stellato *et al.*, 2014 ▸; Botha *et al.*, 2015 ▸) and fixed-target methods (Gati *et al.*, 2014 ▸; Murray *et al.*, 2015 ▸). Serial synchrotron rotation crystallography (SS-ROX; Gati *et al.*, 2014 ▸; Hasegawa *et al.*, 2017 ▸) uses goniometer-based high-dose raster scanning with rotation for data collection. The scheme is useful for microcrystals with a diffracting power that is so weak that low-dose raster scanning cannot specify the crystal positions. The data set for samples with less than 1° rotation is collected from several tens of thousands of crystals that are randomly oriented on the same sample holder.

Although the suitable selection of an experimental scheme for each crystal improves the efficiency of data collection, synchrotron experiments involve additional challenges. Firstly, data collection is time-consuming, particularly for the multiple small-wedge scheme. Specifying crystal positions requires two-dimensional raster scanning with a good signal-to-noise ratio (Cherezov *et al.*, 2009 ▸). For example, cryoloops of 600 µm in size should be scanned using a 10–50 µm square beam for crystal detection. Thus, several hundred or thousand frames, or more, should be collected. Moreover, finding crystals based on the collected frames is also a time-consuming step. To address these issues, a fast raster-scanning system combining a rapid-readout detector and goniometer translation axes has been developed at several microfocus beamlines (Hilgart *et al.*, 2011 ▸; Wojdyla *et al.*, 2016 ▸). Additionally, several rapid spot-finder programs for crystal detection in raster scanning have been reported (Melnikov *et al.*, 2018 ▸). Manual data collection also makes data collection more time-consuming owing to the need to consider the experimental conditions and the input of experimental parameters to the beamline-control software.

State-of-the-art experiments require expert knowledge and skills in data collection. One of the most difficult problems is the estimation of appropriate radiation damage in protein microcrystallography. Several programs, such as *RADDOSE* (Paithankar & Garman, 2010 ▸) and *RADDOSE*3*D* (Zeldin *et al.*, 2013 ▸), have enabled dose estimation before experiments. Nevertheless, experimenters sometimes randomly define exposure conditions without dose estimation, which causes non-equivalent doses among collected data sets. Particularly in merging multiple data sets, non-equivalent doses among data sets may yield confusing results and hamper the correct merging of data sets for structural analysis. Moreover, photoelectron escape from the irradiated crystal volume makes the estimation of radiation damage difficult for modern microfocus beamlines (Nave & Hill, 2005 ▸; Sanishvili *et al.*, 2011 ▸).

Crystal alignment in protein microcrystallography sometimes requires special skills, particularly for helical data-collection schemes. Following 2D raster scanning for the initial specification of crystal position, precise 3D alignment of crystal edges is indispensable. Because the target crystals are smaller, the method is technically more difficult. The experimental protocol is not complex, but requires several scans and judgements for each crystal. The application of routine processes for many crystals often leads to human errors, and this method is time-consuming compared with other schemes. It is also difficult to estimate the appropriate absorbed dose for helical and SS-ROX schemes because each crystal has a different size and is gradually moved during data collection.

Data processing is laborious and time-consuming, particularly when processing a large number of collected data sets. Several program suites for data processing are available, including *XDS* (Kabsch, 1993 ▸, 2010 ▸), *HKL*-2000 (Otwinowski & Minor, 1997 ▸), *iMosflm* (Battye *et al.*, 2011 ▸) and *DIALS* (Sauter *et al.*, 2013 ▸; Winter *et al.*, 2018 ▸). All of these suites require the preparation of input files or input from a GUI interface. If a huge number of data sets have been collected, manual data processing is time-consuming and difficult. Although automatic data-processing pipelines are available to address these issues (Winter, 2010 ▸; Monaco *et al.*, 2013 ▸), the merging of multiple data sets also requires specific processes, such as the grouping of equivalent cells among collected data sets, hierarchical clustering (Giordano *et al.*, 2012 ▸; Foadi *et al.*, 2013 ▸; Santoni *et al.*, 2017 ▸) for isomorphous crystal selection, and breaking the indexing ambiguities (White *et al.*, 2013 ▸; Kabsch, 2014 ▸).

In order to overcome these challenges, *ZOO*, an automatic data-collection system, has been developed at SPring-8. The system is dedicated to protein microcrystallography and enables unattended data collection on the SPring-8 beamlines. The *ZOO* system has automated all conventional goniometer-based data-collection schemes, such as the normal rotation method, helical data collection, multiple small-wedge and synchrotron serial rotation crystallography under cryogenic conditions. Furthermore, *ZOO* can dynamically switch the experimental scheme matched to each crystal by taking crystal sizes and their positional configurations on the cryoloop. In this article, we describe how *ZOO* works and how *ZOO* can contribute to challenging structure analyses.

## 2. The *ZOO* system   

### 2.1. Features   

**Figure 1:**
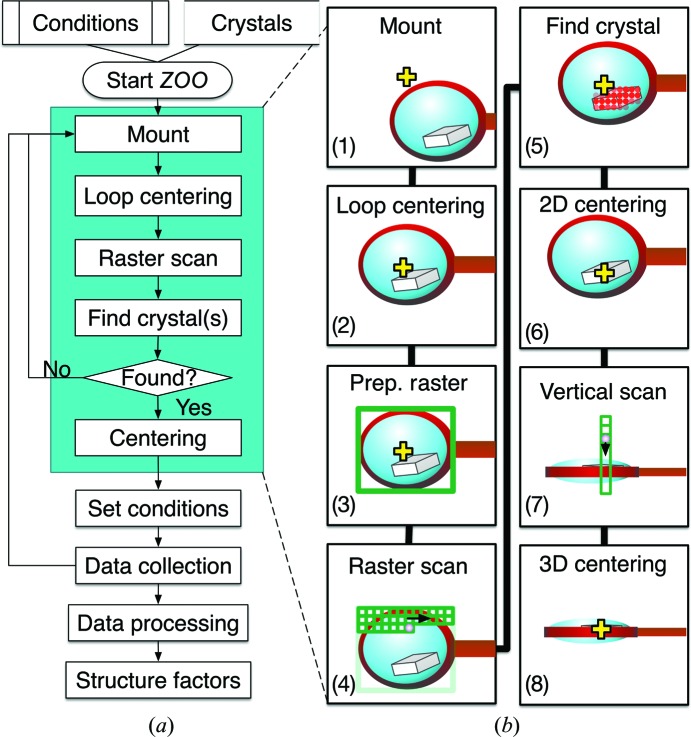
Experimental sequence in *ZOO*. (*a*) The basic sequence for data collection in *ZOO*. The final crystal alignment is based on X-ray diffraction scanning. (*b*) Illustration of the ‘normal rotation scheme’. (1) Sample mounting, (2) loop centering, (3) defining the raster-scan area, (4) low-dose raster scanning, (5) crystal detection, (6) crystal alignment, (7) vertical diffraction scanning at side orientation (90° from the face angle) and (8) crystal alignment. Finally, rotation data is collected from the defined 3D coordinates.

The *ZOO* system automates X-ray diffraction data collection from protein crystals. The experimental sequence automated by *ZOO* is illustrated in Fig. 1 ▸. Experimenters should prepare cryocooled crystals in Unipucks (Crystal Positioning System) and experimental parameters, such as rotation range per crystal, oscillation width, exposure time per image, camera distance and wavelength. A sample list of parameters is given in Table 1 ▸. According to the input information, each sample is sequentially processed.

**Figure 2:**
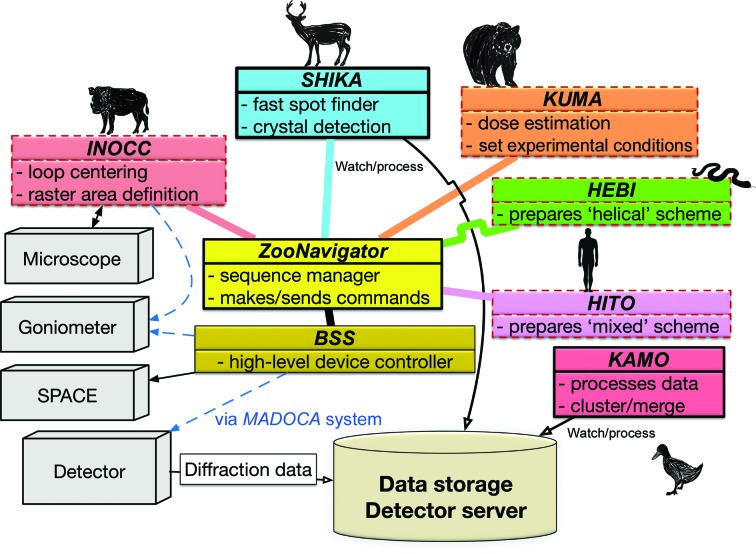
*ZOO* architecture. The system consists of beamline hardware, a beamline-control system and programs for specific tasks. *ZooNavigator* sequentially processes the experimental sequence by communicating with other components. It orders *INOCC* to center the loop after sample mounting by SPACE. *ZooNavigator* receives the raster-scanning area from *INOCC* and starts 2D raster scanning by sending the command to *BSS*. *SHIKA* automatically processes the raster images and *ZooNavigator* receives the heat map and finds crystal positions. *KUMA* returns suitable attenuation conditions from the crystal size and the user-defined conditions given by *ZooNavigator*. In helical and mixed schemes, *ZooNavigator* communicates with *HEBI* and *HITO*. *ZooNavigator* initiates data collection by sending a command to *BSS*. *KAMO* automatically detects the latest data sets on data storage and initiates data processing. Blue dashed lines indicate communications between a program and the SPring-8 device controller *MADOCA*. Programs represented in boxes with dashed lines are Python modules called from *ZooNavigator*.

The architecture of the *ZOO* system is illustrated in Fig. 2 ▸. The system consists of hardware and developed software. The core program, named *ZooNavigator*, a Python script, manages the experimental flow. Each component is sequentially ordered to conduct a specific task or calculation. Each component then returns a response or experimental information to *ZooNavigator*. Finally, *ZooNavigator* combines the received information and the user-defined conditions (for example, a suitable exposure condition is received from *KUMA*) and then sends a command to proceed with the experimental sequence to *BSS*. *BSS* is a standard beamline-control software program utilized for all structural biology beamlines at SPring-8 (Ueno *et al.*, 2005 ▸); this program also works as a server program for a high-level device controller. *ZooNavigator* sends commands, such as ‘exchange sample’ or ‘perform raster scan’, to *BSS*.

*INOCC*, a Python script, is dedicated to automatic loop centering. *INOCC* recognizes the edge of a sample loop from microscope images and aligns its center with the X-ray beam. *ZooNavigator* calls *INOCC* as a Python module and obtains the goniometer *xyz* coordinates of the loop center and the dimensions of the loop area to be covered by raster scanning. After receiving this information, *ZooNavigator* initiates the raster scanning. Details of *INOCC* are described in Section 4.2.

*SHIKA* is a spot-finder program for raster scanning that utilizes a modified version of the *Cheetah* program (Barty *et al.*, 2014 ▸) to adapt to the streaming interface of the EIGER detector. *DISTL* was previously used for spot finding. *SHIKA* automatically detects the raster scan and shows the results on the GUI. Using the number of low-resolution spots up to 5 Å on each image was found to be the most robust method for the detection of crystals. The program finally generates a heat-map file of the scores with the goniometer coordinates. Details are given in Section 4.3.

*KUMA*, a Python script, estimates the exposure conditions based on the absorbed dose estimated using the crystal size and the X-ray beam parameters. The program estimates the absorbed dose for all experimental schemes in *ZOO*. *ZooNavigator* calls *KUMA* as a Python module, and *KUMA* returns the exposure conditions. Details are given in Section 4.4.

*HEBI*, a Python script, prepares helical data collection by analyzing a heat map from *SHIKA*. The program facilitates the complicated procedures of 3D centering of the tiny crystal. Details are given in Section 4.5.

*HITO*, also a Python script, prepares a ‘mixed scheme’ by analyzing a heat map from* SHIKA*. The mixed scheme consists of the ‘multiple small-wedge’ and ‘helical’ schemes. By evaluating the crystal size and the spatial relationship among crystals on the same loop, *HITO* categorizes found crystals into suitable data-collection schemes, such as ‘multiple’ or ‘helical’ schemes. Details are given in Section 4.6.

*KAMO* is an automatic data-processing program package of Python scripts exploiting the *cctbx* library (Grosse-Kunstleve *et al.*, 2002 ▸). *KAMO* utilizes the *XDS* package (Kabsch, 2010 ▸), with *XDS* for the indexing and integration of wedges and *XSCALE* for scaling and merging. *DIALS* is optionally supported for the processing of wedges. *KAMO* helps to decide space-group symmetry, to resolve indexing ambiguities, to perform hierarchical clustering prior to merging and to identify outlier rejections based on the results of *XSCALE* (Yamashita *et al.*, 2018 ▸).

### 2.2. Experimental schemes implemented in *ZOO*   

**Figure 3:**
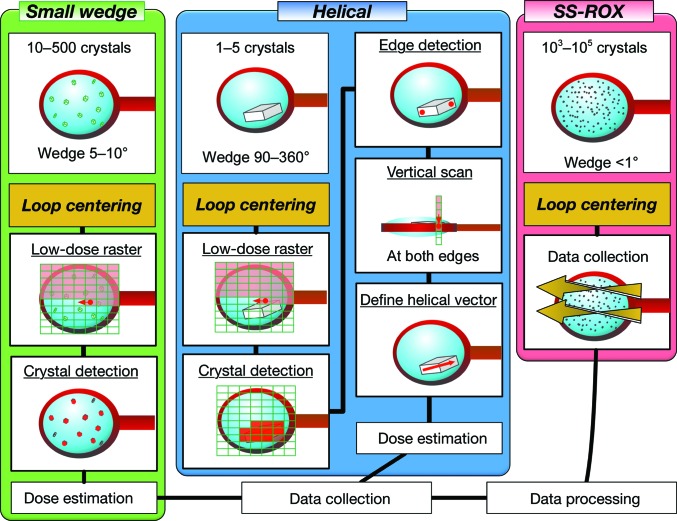
Basic data-collection schemes available in *ZOO*. Small-wedge scheme: small-wedge data collection is applied to crystals found in the 2D raster scan by *SHIKA*. Helical scheme: the X-ray irradiation point on the crystal is kept moving along its longitudinal direction. The scheme requires the initial 2D raster scan for crystal detection. After estimation of the absorbed dose using a ‘helical’ vector on the crystal, shutterless helical data collection is initiated. SS-ROX: the scheme starts high-dose raster data collection with rotation just after loop centering. Details of all schemes, including ‘mixed mode’ (not shown here), are given in Section 2.

Several experimental schemes have been automated using the *ZOO* system. The available schemes are normal rotation (not described here), multiple small-wedge, helical, serial synchrotron rotation crystallography (SS-ROX) and mixed schemes. Users need to select the experimental scheme and conditions before running *ZOO* for their specific purpose. The experimental sequences of each scheme are summarized in Fig. 3 ▸.

#### 2.2.1. Multiple small-wedge scheme   

This scheme is similar to *MeshAndCollect* (Zander *et al.*, 2015 ▸) developed at the ESRF. This sequence is normally applied to a loop with multiple crystals (several to several hundreds). A small-wedge (typically 5–10°) data set is collected from each crystal and merged to complete the data set. Suitable crystal sizes typically range from 5 to 50 µm. *ZOO* automates this scheme as described below (see also Fig. 3 ▸). *INOCC* conducts loop centering and finds a ‘face angle’ at which a face of the loop is perpendicular to the X-rays; the loop area is then defined for raster scanning by *INOCC* (details are given in Section 4.2). 2D raster scanning is typically applied at 50 Hz and 500 µm s^−1^ using a ∼10 × 15 µm (horizontal × vertical) focused beam. *SHIKA* simultaneously finds crystal candidates during the raster scan. After estimating the exposure conditions with *KUMA*, *ZooNavigator* initiates shutterless data collection from each crystal. Typical conditions are shown in Table 1 ▸.

#### 2.2.2. Helical data-collection scheme   

The second scheme automated by *ZOO* is ‘helical data collection’ (Flot *et al.*, 2010 ▸). This scheme is normally applied to crystals larger than the X-ray beam size in order to distribute radiation damage equally over the entire crystal volume. The data-collection scheme is illustrated in Fig. 3 ▸. Sample mounting, loop centering and 2D raster scanning, referred to as common preparation steps, are conducted. After spot finding, *HEBI* analyzes the heat map from *SHIKA* and regards a contiguous area of crystal candidates as a single crystal to determine both ends of the crystal, along with rotation of the axis of a goniometer. *HEBI* first identifies the best grid from the heat map and evaluates the scores of the grids surrounding it. If there are good grids, these are regarded as the same crystal. *HEBI* repeatedly conducts this process. After rotating the goniometer, typically by 90°, vertical diffraction scans are conducted at both crystal edges for 3D centering. Alternatively, precise crystal edge detection can be applied at the face angle using smaller beams before 3D centering. *KUMA* estimates the exposure conditions from the crystal length and other user-defined conditions. *ZOO* initiates shutterless helical data collection.

#### 2.2.3. SS-ROX scheme   

The third scheme is SS-ROX, which is the collection of diffraction images from many crystals (1000–100 000) that are densely mounted on each holder (Gati *et al.*, 2014 ▸; Hasegawa *et al.*, 2017 ▸). Crystals can be rotated during exposure to achieve efficient coverage of reciprocal space. The scheme is effective for crystals with insufficient diffracting power to find their positions using low-dose raster scanning. The data-collection scheme is shown in Fig. 3 ▸. After the common preparation step, *ZooNavigator* receives 2D lengths of the loop at the face angle from *INOCC*. The total oscillation range for each scan line parallel to the rotation axis is estimated based on the horizontal length of the loop, the user-defined scan-step length and the rotation width per frame. *ZooNavigator* orders *BSS* to initiate shutterless rotation data collection with the defined conditions.

#### 2.2.4. Mixed scheme   

The fourth scheme implemented in *ZOO* is a ‘mixed scheme’. This scheme combines the ‘multiple small-wedge’ and ‘helical’ schemes. The scheme is effective when large and small crystals are mounted on the same sample holder. Efficient data collection is achieved by collecting large wedges from larger crystals and small-wedge data from smaller crystals.

**Figure 4:**
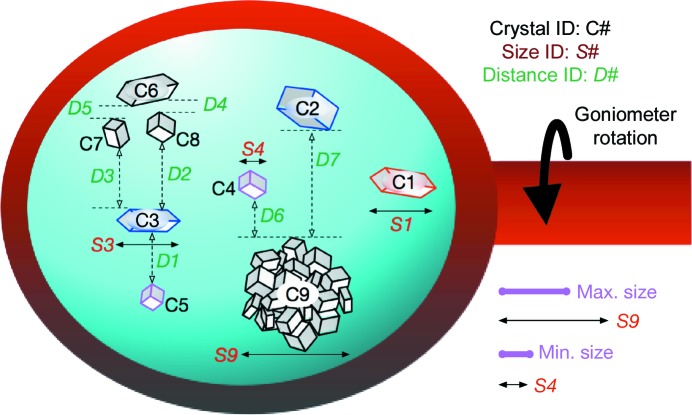
How *HITO* works. Schematic diagram of the spatial relationship analyzed by *HITO*. A cryoloop containing multiple crystals is shown. The rotation axis of the goniometer is horizontal in this figure. *HITO* regards contiguous ‘good’ pixels on the *SHIKA* heat map as individual crystals (C1, C2, C3, …). *HITO* checks overlaps among crystals along the rotation axis. Nonoverlapped crystals are grouped into the ‘helical full rotation’ group (C1). *HITO* calculates the ‘vertical’ distance (D1, D2, …) between vertically overlapped crystals. For example, if this distance is three times longer than the vertical beam size, the crystal is grouped into the ‘partial helical’ group (C2, C3, C4, C5 and C9 at this stage). Others are grouped into ‘clustered crystals’ (C6, C7 and C8). *HITO* calculates the crystal size (*e.g.* S1, S3, S4 and S9). Crystals with smaller sizes along the rotation axis than the ‘minimum size for helical’ are grouped into the ‘small-wedge’ group (C4 and C5). Crystals larger than the ‘maximum size for helical’ are categorized into the ‘clustered’ group (S9).

After the common preparation step, *HITO* analyzes the *SHIKA* heat-map data and determines the data-collection scheme for the identified crystals. Crystal candidates, recognized by *HITO* as with *HEBI*, on the map are grouped into four categories by considering the spatial overlaps of crystals on a sample holder. The first category is ‘helical full rotation’. Crystals in this group have no overlap with other crystals along the rotation axis. Thus, the normal helical scheme can be applied as described in the previous section. The second category is the ‘partial helical’ group, from which relatively smaller wedge data, typically 40–60° per crystal, are collected in a helical manner. Crystals in this group overlap with other crystals along the rotation axis, but the vertical distance is sufficiently long for quasi-3D centering (Fig. 4 ▸). In the quasi-3D centering, when the total rotation per data set is set to 40°, vertical scanning is applied to an edge of the crystal at an angle of −20° relative to the face angle. At another edge of the crystal, an angle of +20° relative to the face angle is applied. Partial helical data collection with 40° rotation is applied to this 3D helical vector. The third category is for the simple ‘small-wedge’ scheme, typically 5–10° per crystal. Crystals in this category are not large enough for helical data collection. The fourth category is ‘clustered’ crystals. Crystals larger than the maximum size for helical data collection are regarded as a bunch of ‘clustered’ crystals. For this group, assuming that the grid on the heat map separated by one pixel vertically and horizontally is a single crystal, ‘small-wedge’ data collection is applied to each crystal. Minimum and maximum crystal sizes for helical data collection, the rotation range for each scheme and the threshold distance for allowing crystal overlap should be given as user-defined parameters. Details of *HITO* are given in Section 4.6. *HITO* passes the crystal list of each category to *ZooNavigator*, which passes the crystal list for ‘helical full rotation’ and ‘partial helical’ to *HEBI* and conducts helical data collection. *ZooNavigator* also initiates multiple small-wedge data collection from ‘small’ and ‘clustered’ crystals.

## 3. Materials   

### 3.1. Beamline   

The *ZOO* system was developed on beamline BL32XU at SPring-8. This beamline is a microbeam beamline that provides a 1 × 1 µm to 10 × 15 µm (horizontal × vertical) beam with a flux density of the order of 10^10^ photons µm^−2^ s^−1^ (Hirata *et al.*, 2013 ▸). This beamline has contributed to the high-resolution structural determination of membrane proteins crystallized by the *in meso* method (Kato *et al.*, 2012 ▸; Doki *et al.*, 2013 ▸; Nishizawa *et al.*, 2013 ▸; Tanaka *et al.*, 2013 ▸; Kumazaki *et al.*, 2014 ▸).

SPACE (Murakami *et al.*, 2012 ▸) is a sample-changer robot developed at SPring-8. It can store eight Unipucks (Crystal Positioning Systems, New York, USA) in the liquid-nitrogen dewar. The ID for each Unipuck should be defined in the SPACE server and the IDs are then referred from *BSS* and *ZooNavigator*. A coaxial microscope (Union Optical, Tokyo, Japan) was designed to visualize micrometre-sized crystals from the same direction with the X-rays. The microscope image server captures the crystal scene when it receives a command via a network socket. The high-precision crystal goniometer (KOHZU Precision, Kanagawa, Japan) consists of 3D translation axes and an air-bearing spindle axis with a sphere of confusion of smaller than 1 µm. The speed of each translation axis, controlled by stepper motors, corresponds to a maximum of 500 µm s^−1^ with a resolution of 0.1 µm. At the first stage in *ZOO* development, the X-ray area detector installed on BL32XU was an MX225HS (Rayonix, Illinois, USA) with 3 × 3 mosaic frame-transfer CCD chips to allow changes in the maximum frame rate, such as 10 Hz with 2 × 2 binning (pixel size 78 µm), suitable for data collection, and 100 Hz with 8 × 8 binning (pixel size 312 µm), which is useful for raster scanning. Currently, an EIGER X 9M detector (Dectris, Switzerland) is installed on the beamline. The detector area and maximum frame rate of the detector are 233.2 × 245.2 mm (width × height) and 238 Hz, respectively. The 4M ROI mode of the detector is sufficient for 5 Å resolution data detection and can be utilized for raster scanning to reduce consumption of data storage. The smaller ROI mode is also effective for reducing the time for spot finding by *SHIKA*.

### 3.2. Crystals for *ZOO* demonstration experiments   

#### 3.2.1. Muscarinic M2 receptor (M2R)   

M2R is a known GPCR. The sample was crystallized by the lipidic cubic phase (LCP) method (Suno, Lee *et al.*, 2018 ▸). The mean crystal size for data collection was ∼30 µm.

#### 3.2.2. Thaumatin   

Thaumatin (catalog No. 201-11351; Wako Pure Chemical Industries, Osaka, Japan) was crystallized by hanging-drop vapor diffusion. Droplets were composed of 2 µl protein solution and 2 µl seeding solution consisting of ground seeding crystals, 50 m*M* HEPES pH 7.5, 1.2 *M* potassium/sodium tartrate. Reservoirs were composed of 50 m*M* HEPES pH 6.5–8.0, 0.70–0.95 *M* potassium/sodium tartrate. The crystals were transferred to a cryoprotectant solution consisting of 50 m*M* HEPES pH 7.5, 1.2 *M* sodium chloride, 30% glycerol before cryocooling. The mean crystal size for data collection was 20–30 µm.

#### 3.2.3. Thermolysin   

Thermolysin (catalog No. T7902-250MG; Sigma–Aldrich, Missouri, USA) was crystallized by sitting-drop vapor diffusion. The protein was dissolved to a concentration of 100 mg ml^−1^ in a buffer solution consisting of 50 m*M* Tris–HCl, 45% DMSO, 2.5 *M* caesium chloride. After placing 4 µl of the solution into each well of a 96-well sitting-drop plate, the wells were sealed with reservoir composed of 0.1–1.0 *M* sodium chloride solution. The crystals were cryocooled after bathing in a cryoprotectant solution consisting of 50 m*M* Tris–HCl, 45% DMSO, 2.5 *M* caesium chloride with 25% glycerol for 1 min.

#### 3.2.4. Lysozyme   

Lysozyme (catalog No. E89201; Seikagaku, Tokyo, Japan) was crystallized by hanging-drop vapor diffusion. The protein was dissolved in 50 m*M* acetate buffer pH 4.5 to a concentration of 25 mg ml^−1^. The droplets were composed of 5 µl protein solution and 5 µl seeding solution consisting of ground seeding crystals, 50 m*M* acetate buffer pH 4.5, 1.6 *M* sodium chloride. The reservoirs were composed of 50 m*M* acetate buffer pH 4.4–4.7, 0.90–1.15 *M* sodium chloride. The crystals were cryocooled after transfer to a cryoprotectant solution consisting of 50 m*M* acetate buffer pH 4.5, 1.4 *M* sodium bromide, 25% glycerol before cryocooling for ∼20 s. Crystal sizes for data collection ranged from 10 to 150 µm.

## 4. Components of *ZOO*   

### 4.1. Data-acquisition system for rapid raster scanning and shutterless measurement   

The data-acquisition system for rapid raster scanning and shutterless measurement was implemented by combining a high-speed area detector and a high-precision goniometer with a precise timing-control system.

The procedure for raster scanning consisted of the iterative acquisition of a series of diffraction images from the area detector with the periodic operation of exposure and readout cycles triggered by the external electrical signal, accompanied by the linear translation of the sample at a constant speed along the specified grid array. The X-ray beam shutter was kept open during the scanning (shutterless data acquisition).

Since it is necessary to reposition the identified crystals precisely at the X-ray beam for effective raster scanning and subsequent data collection, precise synchronization between the goniometer motion and the electrical trigger signal for the detector is essential. Synchronization among these devices was made possible by installing a Blanc8 multifunctional control unit (Ishii & Ohata, 2009 ▸) developed at the JASRI Controls and Computing Division at SPring-8. Blanc8 consists of a COM Express motherboard and riser card for PCIe and PCI slots. The electrical trigger signal for the area detector and X-ray beam shutter, and the control signal for the crystal goniometer axes, are provided via a commercial motion-controller board (Interface, Hiroshima, Japan). The operating system of Blanc8 is real-time Linux, on which the server process of the SPring-8 standard device-control command protocol of *MADOCA* (Tanaka *et al.*, 1995 ▸) is run. Hardware control from the beamline-control software *BSS* (Ueno *et al.*, 2005 ▸), to which the condition of raster scanning is registered during the course of automatic data collection by the *ZOO* system, is available by communicating with the server process through a network socket.

The raster-scan range defined based on the sample dimensions was divided into 2D grid arrays in the horizontal and vertical directions, perpendicular to the X-ray beam. The grid size was normally set to the same size as the X-ray beam size: typically 10 µm for both directions. The detector frame rate was normally set to 50 Hz. A raster scan was conducted by executing a multiple scan series along the horizontal or vertical grid arrays by changing the start and end points one by one. For each scan, the goniometer translation speed was calculated and the timing of the axis motion was adjusted by delay parameters for each axis within microseconds. The timing jitter of the synchronization for each linear scan was less than 2 ms, which corresponds to a positional error of less than 1 µm under the default conditions described above; this was sufficiently smaller than the grid size.

### 4.2. *INOCC*: loop centering   

**Figure 5:**
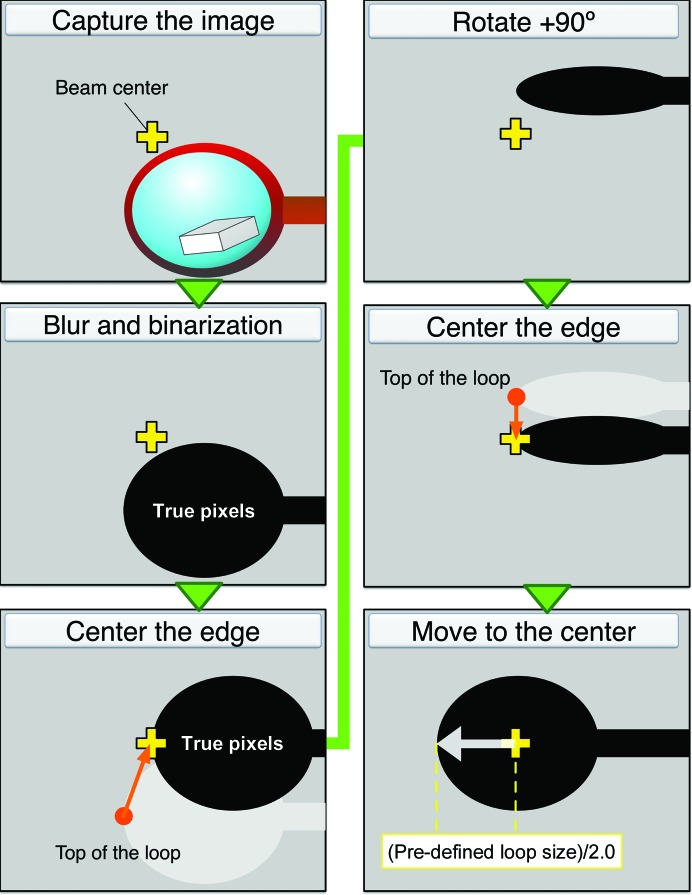
Loop centering by *INOCC*. After blurring and binarization of the captured image with *OpenCV* (Bradski, 2000 ▸), the program recognizes the left edge of the loop and translates it to the X-ray irradiation point at rotation angles of 0°, 45°, 90° and 135°. After convergence of the process, the face angle where the face of the loop is perpendicular to the X-rays is found. Finally, the program moves the loop to the left by a half-length of the user-defined loop size along the rotation axis.

Three major functions were implemented. Firstly, the center of gravity of the loop shape is translated to the beam center. Secondly, the program finds the orientation in which the largest face of the cryoloop is perpendicular to the X-ray path. This angle is referred to as the ‘face angle’. Finally, at the face angle *INOCC* recognizes the edge of the cryoloop and defines the raster area as a rectangular shape enclosing the loop (Fig. 5 ▸).

*INOCC* was developed using Python2.6 and *OpenCV* v.2.4 (Bradski, 2000 ▸). This program communicates with the beamline-control system (*MADOCA* system) to control three translational motorized axes and the rotation axis of the goniometer, which also communicates with the image server of the on-axis microscope. Initially, the background image is subtracted from the captured image of a loop. After generating a grayscaled image, *INOCC* applies Gaussian blurring and binarization using the cvtColor, GaussianBlur and threshold functions in *OpenCV*. In the binarized image, pixels with nonzero values show the existence of an object, referred to here as ‘true pixels’.

The first step in loop centering is to find the cryoloop. *INOCC* analyzes binarized images at rotation angles of 0°, 45°, 90° and 135°. If there are no true pixels at any angle, the goniometer horizontal axis is translated to 1.5 times longer than the horizontal width of the captured image (2 mm at the BL32XU beamline). *INOCC* iterates this process twice.

When the binarized image includes many true pixels, *INOCC* regards the leftmost pixel as the top of the cryoloop. Goniometer axes are translated to match the top of the cryoloop to the beam center in the horizontal direction. For the vertical direction, the center of gravity of true pixels is matched to the beam height. *INOCC* repeats this process at rotation angles of 0°, 45° and 90°.

During the next step, the face angle is searched for 2D raster scanning. *INOCC* counts true pixels in binarized images at rotation angles of 0°, 45°, 90° and 135°. By fitting the number of true pixels at these angles to the cosine function, the program finds the angle at which the loop looks smallest from the X-ray viewpoint. The face angle is 90° from the resulting angle. After edge centering at the face angle, *INOCC* makes a square circumscribing the edge of the loop. The horizontal and vertical lengths of the square are divided by the user-defined horizontal and vertical beam sizes of the 2D raster scan, respectively. *INOCC* passes this information to *ZooNavigator*. Users can define the utilized loop size to limit the horizontal length for 2D raster scanning. The vertical scan range is automatically defined by the found edge.* ZooNavigator* saves the goniometer coordinates of the loop; the next loop is then moved to the saved position to decrease the time spent finding the loops. The average time for loop centering is around 40 s.

### 4.3. *SHIKA*: spot finding   

**Figure 6:**
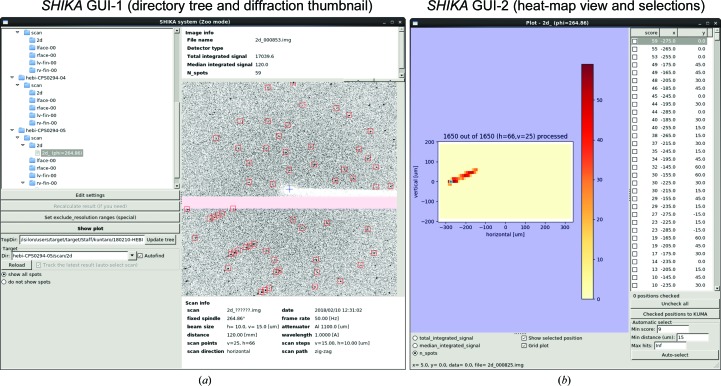
*SHIKA* GUI. Elements of the GUI for the *SHIKA* front end initially developed for manual data collection. *SHIKA* analyzes each raster-scanned image up to 5 Å resolution so as to avoid false-negative detection of the ice-ring (∼3.7 Å) and lipid-ring (∼4.5 Å) patterns of *in meso* phase crystals (Ueno *et al.*, 2016 ▸). In the thumbnail in (*a*), red rectangles follow the identified diffraction peaks. (*b*) The entire view of the heat map is available in another window. This heat map shows 20 × 20 × 100 µm needle-shaped thermolysin crystals. Users can check the diffraction pattern in the thumbnail window by clicking a grid on the heat map.

*SHIKA* was developed to analyze the raster-scan images obtained by *BSS*. *SHIKA* consists of three programs: a front-end GUI (Fig. 6 ▸), a back end and *Cheetah* clients. Multiple *Cheetah* clients are executed to process the detector images received via ZeroMQ in parallel. In the *Cheetah* client, the *peakfinder*8 algorithm in *Cheetah* (Barty *et al.*, 2014 ▸) is used for spot detection, and spot intensities and coordinates are passed to the *SHIKA* back end together with the generated thumbnail detector images. The *SHIKA* back end serializes the results received via ZeroMQ and writes the SQLite database file shika.db, 10 × 10 tiled thumbnail images in JPEG format and the resulting file summary.dat that describes grid coordinates and scores into the directory where raster scanning was performed. The file is utilized as a *SHIKA* heat map to define crystal positions. For EIGER detectors, *Cheetah* clients directly connect to the EIGER DCU server, which streams frame data in PUSH mode from ZeroMQ. Alternatively, the *SHIKA* back end detects detector-image files appearing in the file system and streams the file information to *Cheetah* clients. The *SHIKA* GUI reads shika.db and shows the heat map of spot numbers. By clicking a grid on the heat map, a thumbnail image is shown with the picked spot positions highlighted. Users can choose positions arbitrarily or use a function to automatically select higher score positions with a specified minimum distance. Selected positions can be transferred to *KUMA* using the XML-RPC server.

### 4.4. *KUMA*: suggestion of exposure conditions   

*KUMA* was first developed to estimate the absorbed dose for helical data collection (Hirata *et al.*, 2016 ▸) and can be used for dose estimation in all experimental schemes in *ZOO*. *KUMA* currently exploits *RADDOSE* (v.2; Paithankar & Garman, 2010 ▸) to calculate an absorbed dose from a specified crystal and X-ray parameters for nonhelical data-collection schemes, such as multiple small-wedge and normal schemes. Particularly for helical and SS-ROX schemes, estimating the correct absorbed dose is complicated because the crystal volumes illuminated by X-rays are normally overlapped in neighboring frames. All of the experimental parameters, such as beam size, X-ray energy, exposure time per frame, crystal length and rotation range, need to be carefully taken into consideration. *KUMA* predicts the absorbed dose on crystals and proposes suitable exposure conditions from user-defined parameters. Its function is similar to that of *RADDOSE*-3*D* (Zeldin *et al.*, 2013 ▸). A major difference between *KUMA* and *RADDOSE*-3*D* is that *KUMA* estimates the dose based on experimental data for the propagation length of radiation damage in frozen protein crystals (Hirata *et al.*, 2016 ▸). By default, *KUMA* suggests exposure conditions with an absorbed dose of 10 MGy.

### 4.5. *HEBI* (for helical data collection)   

*HEBI* interprets the heat map of 2D raster scanning at the face angle and defines the 3D vector for helical data collection. On the map, *HEBI* regards many contiguous grids with higher scores than the user-defined threshold as individual crystals. The program does not deal with crystal overlaps along the rotation axis. For each crystal, *HEBI* selects the leftmost and rightmost edges along with the rotation axis. At the left edge, *HEBI* rotates the goniometer to the start angle of helical data collection. If the total oscillation exceeds 180° then it is set to −90° from the face angle. *HEBI* conducts ‘vertical’ 1D scanning and identifies the best grid from the *SHIKA* heat map. The same scheme is also applied to the right edge at the end angle of data collection. If the total oscillation exceeds 180°, it is set to +90° from the face angle. Consequently, *HEBI* determines the 3D coordinates of both crystal edges, referred to as the ‘helical vector’, as goniometer coordinates. The program orders *KUMA* to estimate the suitable exposure conditions from each helical vector and passes all information to *ZooNavigator*. After receiving the required information from *HEBI*, *ZooNavigator* initiates helical data collection. *HEBI* requires the user-defined parameters listed in Table 1 ▸. If the crystals are smaller than 30 µm, precise crystal edge detection should be performed by 2D raster scanning with a small beam (*e.g.* 1 × 5 µm) at the face angle before vertical scan sequences.

### 4.6. *HITO* (for the ‘mixed scheme’)   

*HITO* interprets the heat map of 2D raster scanning at the face angle and prepares ‘mixed-scheme’ data collection for a loop containing multiple small and large crystals. *HITO* automatically selects a suitable scheme for each crystal by interpreting the spatial relationships of crystals on the loop.

*HITO* first analyses the *SHIKA* heat map. The program regards contiguous pixels with better scores than the user-defined threshold on the heat map as individual crystals. *HITO* then checks overlaps among crystals along the rotation axis. Nonoverlapped crystals are grouped into the ‘helical full rotation’ group (C1 in Fig. 4 ▸). For overlapped crystals, *HITO* calculates the ‘vertical’ distance (D1, D2, … in Fig. 4 ▸). If this distance is three times longer than the vertical beam size for data collection, the crystal is grouped into the ‘partial helical’ group (C2, C3, C4, C5 and C9 at this stage). Others are grouped into ‘clustered crystals’ (C6, C7 and C8). *HITO* calculates the crystal size (*e.g.* S1, S3, S4 and S9), and crystals with smaller sizes along the rotation axis than the ‘minimum size for helical’ are grouped into the ‘small-wedge’ group (C4 and C5). Crystals larger than the ‘maximum size for helical’ are categorized into the ‘clustered’ group (S9). *HITO* requires user-defined parameters, such as the minimum and maximum crystal sizes for helical data collection and wedge sizes for both the ‘helical full rotation’ and ‘partial helical’ schemes.

## 5. Examples of data collection with *ZOO*   

### 5.1. Multiple small-wedge scheme   

To demonstrate the feasibility of small-wedge data collection for *de novo* structure determination, we applied the *ZOO* scheme to the SAD phasing of mercury-bound M2 receptor crystals. The 2D raster scanning was performed using a 10 × 10 µm (horizontal × vertical) focused beam (6 × 10^10^ photons per frame). Automatic data collection was performed at a wavelength of 1.0000 Å using 32 loops. A total of 671 crystals were identified, and 5.0° of data were collected from each (see Table 2 ▸). Moreover, 547 data sets were indexed and integrated with *XDS* through *KAMO*. Of these, 529 data sets had consistent unit-cell parameters, which were subjected to clustering and merging with outlier rejection. Determination of the heavy-atom sites, phasing and phase improvement were performed with *SHELXC*, *SHELXD* and *SHELXE* (versions 2013/2, 2013/1 and 2014/4, respectively; Sheldrick, 2010 ▸). The coordinates of two Hg atoms per asymmetric unit were identified using *SHELXD* with a CC_all_ of 32.3% and a CC_weak_ of 17.7%. Using the heavy-atom sites, phasing and phase-improvement calculations were then carried out with *SHELXE*. The *SHELXE* procedure of density modification and polyalanine auto-tracing resulted in a mean figure of merit (FOM) of 0.643 and modeled 253 residues with CC = 45.85%. A total of 401 min were required to collect all data sets from 32 loops with the MX225HS detector in 2015. The *ZOO* system can currently process each loop containing 30 crystals within ∼7 min using the EIGER X 9M detector. The PDB accession code of the final structure is 5yc8. The raw diffraction images are available in the Zenodo data repository (https://doi.org/10.5281/zenodo.1094808).

### 5.2. Helical scheme   

We evaluated the ‘helical data-collection scheme’ of *ZOO* using thermolysin crystals (Table 2 ▸). The crystal size was approximately 30 × 30 × 220 µm. Low-dose 2D raster scanning was performed using a 10 × 15 µm (horizontal × vertical) beam with a transmission of 1.8% and at 50 Hz (3 × 10^8^ photons per frame). After determination of the 3D helical vector from vertical scans at both crystal edges using a 2 × 15 µm (horizontal × vertical) beam, *KUMA* was used to determine the exposure conditions from the helical vector. Helical data collection was conducted using an X-ray wavelength of 1.0000 Å. A beam size of 2 × 15 µm (horizontal × vertical) was utilized for data collection. The user-defined dose limit was set to 8 MGy for helical data collection. The structure was solved by rigid-body refinement using the isomorphous thermolysin structure (PDB entry 1kei; M. Senda, T. Senda & S. Kidokoro, unpublished work). After a few cycles of manual inspection using *Coot* and automated refinement with *phenix.refine*, refinement converged with *R*_work_ and *R*_free_ values of 0.1658 and 0.1849, respectively. The raw diffraction images are available in the Zenodo data repository (https://doi.org/10.5281/zenodo.1209098).

### 5.3. SS-ROX scheme   

Using tetragonal thaumatin crystals, we collected rotational serial snapshots of 1° per frame at 30 Hz using a 10 × 18 µm beam without any attenuation (6.2 × 10^12^ photons per frame; Table 2 ▸). Of 23 586 snapshots collected from nine loops using an MX225HS detector, 3263 snapshots were identified as hits based on the criterion of more than nine spots in the lower resolution range picked by *SHIKA*. The absorbed dose for data collection was set to 8 MGy. Of these, 2154 snapshots were indexed and integrated using *XDS* through *kamo.single_images_integration* and merged by averaging all observations to 1.4 Å in the Monte Carlo fashion using *kamo.merge_single_integrated_frames*. The structure was solved by rigid-body refinement using the isomorphous thaumatin structure (PDB entry 1rqw; Q. Ma & G. M. Sheldrick, unpublished work). After a few cycles of manual inspection using *Coot* and automated refinement with *phenix.refine*, refinement converged with *R*_work_ and *R*_free_ values of 0.1906 and 0.2018, respectively. The raw diffraction images are available in the Zenodo data repository (https://doi.org/10.5281/zenodo.1209491).

### 5.4. Mixed scheme   

The mixed scheme in *ZOO* was evaluated using lysozyme crystals derivatized with bromide (Table 2 ▸). *ZOO* processed a loop containing multiple crystals of various sizes. The minimum and maximum crystal sizes for the helical schemes were set to 20 and 100 µm, respectively. *HITO* categorized crystals on the loop and *ZOO* successfully collected a 60° ‘helical full rotation’ wedge from one crystal, 40° ‘partial helical’ wedges from seven crystals and 5° ‘small wedges’ from 60 crystals. All data sets were processed automatically and successfully merged with *KAMO*. Determination of the heavy-atom sites, phasing and phase improvement was performed with the *SHELXC*, *SHELXD* and *SHELXE* programs (versions 2016/1, 2013/2 and 2018/1, respectively; Sheldrick, 2010 ▸). The coordinates of five Br atoms per asymmetric unit were identified using *SHELXD* with a CC_all_ of 29.9% and a CC_weak_ of 16.8%. Using the heavy-atom sites, phasing and phase-improvement calculations were then carried out with *SHELXE*. The *SHELXE* procedure of density modification and polyalanine autotracing resulted in a mean FOM of 0.601 and modeled 112 residues with CC = 34.36%. The initial model was loaded into *Buccaneer* (Cowtan, 2006 ▸) in *CCP*4 for automatic protein chain building. After several cycles of manual inspection using *Coot*, refinement converged with *R*_work_ and *R*_free_ values of 0.1990 and 0.2204, respectively. The raw diffraction images are available in the Zenodo data repository (https://doi.org/10.5281/zenodo.1208078).

## 6. Conclusion   

In this report, we describe an automated data-collection system, *ZOO*, and evaluate its ability to analyze various experimental schemes. The *ZOO* system successfully automated all possible goniometer-based data-collection protocols. Moreover, the *ZOO* system dramatically shortened the time needed for data collection by using the ‘fast raster scan’ system and the fast spot-finder program *SHIKA*. Additionally, *ZooNavigator* smoothly connected the experimental sequence without any time gaps; for example, time to input commands for the beamline GUI. *KUMA* also reduces the time required for considering suitable exposure conditions.

The *ZOO* system has dramatically reduced the requirement for expert knowledge and skills in data collection. In particular, *KUMA* estimates suitable exposure conditions from user-defined doses for all data-collection schemes in *ZOO*. *HEBI* permits users to avoid the complicated procedures used to align 3D helical vectors with X-rays. *HITO* automatically selects a suitable data-collection scheme for each crystal by analyzing the spatial relationships among crystals on the same loop. Moreover, the automatic data-processing software program *KAMO* reduces the labor and the time required to process huge numbers of collected data sets. The system has an option to conduct automatic clustering and merging. *KAMO* immediately shows these results on a web browser. Users can begin structure analysis using reflection files from better resulting clusters with less crystallographic knowledge. Thus, the *ZOO* system dramatically reduces the amount of time that a researcher must spend working on the beamline instrument and eliminates human errors during data collection.

Finally, the *ZOO* system can control the quality of the collected data sets. Firstly, the system avoids data collection from crystals with low diffracting power by using the results of low-dose raster scanning. Additionally, *KUMA* dramatically reduces the chance of severe radiation damage to data sets. Furthermore, *KAMO* conducts hierarchical clustering analysis and enables better selection of the data sets to be merged. Hence, crystals with very low quality do not contribute to the resulting data set. Enhanced experimental efficiency and data quality accelerate the accumulation of better data sets during the limited machine time. This accumulation makes it much easier to select better data sets for merging with sufficient completeness. Merging many data sets enhances the signal-to-noise ratio and gives higher resolution data for structural analysis. The combined beneficial effects of the *ZOO* system have accelerated the high-resolution structural analysis of challenging samples (Shihoya *et al.*, 2017 ▸; Suno, Kimura *et al.*, 2018 ▸; Abe *et al.*, 2017 ▸). Accordingly, we concluded that automated data collection dramatically improves the data quality based on quantitative changes in conventional goniometer-based data collection at the synchrotron facility.

## 7. Future work   

Currently, *ZOO* continues data collection until it is stopped or until all user-defined experiments are finalized. When the time for data collection is not sufficient, all data collection cannot be completed. Thus, it is preferable to detect ‘data completion’ for ongoing samples in *ZOO* experiments. For example, if *ZooNavigator* can detect data completion by communicating with *KAMO*, it is possible to automatically stop the measurements and start data collection for the next sample. Although *HITO* can automatically select a data-collection scheme according to the crystal size and the spatial relationships among the crystals, it still requires user-defined parameters. Future development involves the implementation of more intelligent functions, such as the distinction of each crystal orientation or crystal overlap (Melnikov *et al.*, 2018 ▸), in *ZOO* to eliminate user-defined parameters.

## 8. Availability   

The source code for the *Cheetah* client in *SHIKA* is available under a GPL license at the GitHub website (https://github.com/keitaroyam/cheetah/tree/eiger-zmq). The automatic data-processing system *KAMO* is available under the new BSD license at the GitHub website (https://github.com/keitaroyam/yamtbx), where the remaining parts of *SHIKA* will also become available in the near future.

## Supplementary Material

Raw diffraction images of human muscarinic acetylcholine receptor. URL: https://doi.org/10.5281/zenodo.1094808

Thermolysin dataset collected with the helical scheme implemented in HEBI in the ZOO system. URL: https://doi.org/10.5281/zenodo.1209098

Thaumatin data collected with the SSROX scheme implemented in ZOO system. URL: https://doi.org/10.5281/zenodo.1209491

## Figures and Tables

**Table 1:** List of parameters required for *ZooNavigator* The values are typical conditions for multiple small-wedge data collection by *ZOO*.

Parameter name	Example for ‘small-wedge’ scheme
Mode	Small wedge
Puck ID	CPS1974
Pin ID	1–16
Total oscillation per wedge (°)	5.0
Oscillation width (°)	0.1
Exposure time for raster scan (s)	0.02†
Photons per frame in 2D raster scan	10^10^ †
Beam size (µm)	10 (horizontal) × 15 (vertical)†
Dose per wedge (MGy)	10.0
Exposure time for data collection (s per frame)	0.05
Camera distance (mm)	200
Loop size (µm)	600
No. of spots for good crystals	15–100‡
Data directory	/data/here/
Data name	multi
Maximum No. of data collections per loop	100

† Typical value for finding crystals for challenging samples, such as GPCRs.

‡ Lower and upper thresholds for *SHIKA* can be set. If the number of spots exceeds 100, the grids are neglected in this case.

**Table 2:** Data-statistics table for example data collections with the *ZOO* system

	M2R (SAD)	Thaumatin	Thermolysin	Lysozyme (Br-SAD)
Collection scheme	Small wedge	SS-ROX	Helical	Mixed
Wavelength (Å)	1.0000	1.0000	1.0000	0.9000
Space group	*P*2_1_	*P*4_1_22	*P*6_1_22	*P*4_3_2_1_2
*a*, *b*, *c* (Å)	46.5, 59.0, 89.2	58.1, 58.1, 150.4	93.0, 93.0, 129.5	79.1, 79.1, 37.5
α, β, γ (°)	90, 98.9, 90	90, 90, 90	90, 90, 120	90, 90, 90
Beam size (height × width) (µm)	10 × 10	10 × 18	2 × 15	10 × 15
Dose per wedge (MGy)	12	8	8	8–10
No. of loops	32	9	1	1
Total collection time (min)	401	89	6	21
No. of collected wedges	671	3263°	1	60° × 1, 40° × 7†, 5° × 60
No. of used wedges	459	2154°	1	60° × 1, 40° × 7†, 5° × 60
Resolution range (Å)	49.1–2.50 (2.59–2.50)	50.0–1.40 (1.45–1.40)	40.3–1.46 (1.51–1.46)	39.6–1.50 (1.55–1.50)
Completeness (%)	99.98 (99.88)	100 (100)	99.76 (98.83)	100 (100)
Multiplicity	24.2 (23.7)	340 (310)	19.7 (20.0)	20.3 (19.0)
*R*_meas_	0.506 (6.176)	n/a	0.118 (1.74)	0.150 (2.45)
*R*_p.i.m._	0.101 (1.252)	n/a	0.026 (0.382)	0.032 (0.541)
〈*I*/σ(*I*)〉	10.2 (1.4)	6.5 (1.5)	14.5 (1.44)	13.8 (1.46)
CC_1/2_	0.996 (0.602)	0.984 (0.523)	0.998 (0.687)	0.999 (0747)
CC_ano_	0.20	n/a	n/a	0.289
Refinement				
*R*_work_	0.237	0.191	0.166	0.199
*R*_free_	0.270	0.202	0.185	0.221
No. of atoms				
Protein	3042	1564	2496	1001
Water	26	184	293	152
Ligand	22	16	5	
Average *B* factor (Å^2^)				
Protein	63.9	14.9	22.7	23.6
Water	40.4	26.5	37.8	33.97
Ligand	39.5	15.6	19.7	
R.m.s.d. from ideal				
Bond lengths (Å)	0.002	0.006	0.007	0.006
Bond angles (°)	0.491	1.178	0.92	0.84
Ramachandran plot				
Favored (%)	96.83	96.82	96.5	97.64
Allowed (%)	2.91	3.18	3.50	2.36
Outlier (%)	0.26	0.0	0.0	0.0

† Helical full rotation, 60°; partial helical, 40°; small wedge and cluster, 5°.
